# Internet gaming disorder, aggression and psychological distress in young adults

**DOI:** 10.1192/j.eurpsy.2022.349

**Published:** 2022-09-01

**Authors:** B.R. Maia, G. Reis

**Affiliations:** Universidade Católica Portuguesa, Faculty Of Philosophy And Social Sciences, Braga, Portugal

**Keywords:** internet gaming disorder, aggressiveness, young adults, psychologica distress

## Abstract

**Introduction:**

Internet gaming has become a topic of interest since it has positive but also negative effects.

**Objectives:**

To explore the relationship between internet gaming, aggression and psychological distress in young adults.

**Methods:**

229 Portuguese subjects (55.5% females), with a mean age of 21.13 years old (*SD* = 2.075, range: 18-29) filled in the Internet Gaming Disorder Scale-Short Form, the Buss-Perry Aggression Questionnaire, and the Depression, Anxiety and Stress Scales-21.

**Results:**

The total score of internet gaming was of 15.90 (SD=6.32), 79.9% (n=183) of the sample used to play videogames and 24.5% (n=56) spent more than ten hours playing a week. Internet gaming was correlated with physical aggression (r=.23**), anger (r=.31**) and hostility (r=.35**); and with depression (r=.36**), anxiety (r=.28**), and stress (r=.31**). A Mann Whitney U test revealed significant differences in internet gaming disorder levels of males (Md=130.75, n=102) and females (Md=102.35, n=127), U=4871.000 z=-3.232, p=.001, r=4.49.

**Conclusions:**

Internet gaming disorder is associated with aggression and psychological distress, and males presented higher internet gaming disorder levels. Future studies are needed to explore the bidirectional relationships between gaming disorder, aggression and psychological distress.

**Disclosure:**

No significant relationships.

